# Different particle flow patterns from the airways after recruitment manoeuvres using volume-controlled or pressure-controlled ventilation

**DOI:** 10.1186/s40635-019-0231-8

**Published:** 2019-03-13

**Authors:** Ellen Broberg, Leif Pierre, Mohammed Fakhro, Lars Algotsson, Malin Malmsjö, Snejana Hyllén, Sandra Lindstedt

**Affiliations:** 1 Department of Cardiothoracic Anaesthesia and Intensive Care, Skane University Hospital, Lund University, Lund, Sweden; 2 Department of Cardiothoracic Surgery and Transplantation, Skane University Hospital, Lund University, Lund, Sweden; 3 Wallenberg Center for Molecular Medicine, Lund University, Lund, Sweden; 4 Department of Ophthalmology, Skane University Hospital, Lund University, Lund, Sweden

## Abstract

**Objectives:**

Noninvasive online monitoring of different particle flows from the airways may serve as an additional tool to assess mechanical ventilation. In the present study, we used a customised PExA, an optical particle counter for monitoring particle flow and size distribution in exhaled air, to analyse airway particle flow for three subsequent days. We compared volume-controlled ventilation (VCV) and pressure-controlled ventilation (PCV) and performed recruitment manoeuvres (RM).

**Methods:**

Six animals were randomised into two groups: half received VCV before PCV and the other half received PCV before VCV. Measurements were taken daily for 1 h in each mode during three subsequent days in six fully anaesthetised domestic pigs. A RM was performed twice daily for 60 s at positive end-expiratory pressure (PEEP) of 10, 4 breaths/min and inspiratory-expiratory ratio (I:E) of 2:1. Measurements were taken for 3 min before the RM, 1 min during the RM and for 3 min after the RM. The particle sizes measured were between 0.48 and 3.37 μm.

**Results:**

A significant stepwise decrease was observed in total particle count from day 1 to day 3, and at the same time, an increase in fluid levels was seen. Comparing VCV to PCV, a significant increase in total particle count was observed on day 2, with the highest particle count occurring during VCV. A significant increase was observed comparing before and after RM on day 1 and 2 but not on day 3. One animal developed ARDS and showed a different particle pattern compared to the other animals.

**Conclusions:**

This study shows the safety and useability of the PExA technique used in conjunction with mechanical ventilation. We detected differences between the ventilation modes VCV and PCV in total particle count without any significant changes in ventilator pressure levels, FiO_2_ levels or the animals’ vital parameters. The findings during RM indicate an opening of the small airways, but the effect is short lived. We have also showed that VCV and PCV may affect the lung physiology differently during recruitment manoeuvres. These findings might indicate that this technique may provide more refined information on the impact of mechanical ventilation.

## Background

Mechanical ventilation is a lifesaving treatment that also can inflict lung damage such as acute lung injury (ALI) and ventilator-induced lung injury (VILI). The main concern is avoiding lung injury, both barotrauma (pressure-induced injury) and volume-trauma, in patients treated with mechanical ventilation. To reduce the risk of ALI and VILI, previous studies have shown that pressure-controlled ventilation (PCV) might be preferable to volume-controlled ventilation (VCV) but the studies are not conclusive [1–4]. During mechanical ventilation, recruitment manoeuvres (RM) are frequently used, but their efficacy and risks is an area of intense debate, especially in the most vulnerable groups of patients, such as those with acute respiratory distress syndrome (ARDS). The optimal RM has not yet been determined, nor has the optimal time during the course of mechanical ventilation that the RM should be performed [5–7]. In this study, we have focused on comparing VCV and PCV, along with comparing a gentle RM used during both VCV and PCV.

In clinical practice, airways are monitored by measuring pressure, volume and airflow. We have earlier described a new method to monitor airways noninvasively by analysing different particle flow and size distribution online during mechanical ventilation using an optical particle counter in a customised PExA device [8]. This study has been performed over a period of 3 days to establish the feasibility and safety of the PExA method to be used in conjunction with mechanical ventilation.

Exhaled breath particles (EBPs) are believed to be formed from the respiratory lining fluid in the distal parts of the lung during the opening and closing of the small airways and have been explored as potential markers for different lung diseases in spontaneously breathing patients [9–11]. Monitoring airways by analysing different particle flow online from the small airways may provide real-time insight into the effects of mechanical ventilation before changes in conventional parameters can be detected such as peak pressures, compliance and increased need of oxygen.

Different ventilation strategies during and after abdominal surgery have been studied, and the evidence suggests a protective ventilation with lower tidal volumes (6–8 ml/kg) and low levels of PEEP leading to fewer postoperative lung complications [12–15]. In this study, the animals underwent an initial abdominal surgery procedure to mimic a possible scenario in an intensive care unit in order to explore the impact of ventilation type by comparing optical particle counterflow and size patterns for two different ventilation modes, VCV and PCV, while using protective ventilation with tidal volumes of 6–8 ml/kg and PEEP of 5 during both modes.

Importantly, this study establishes the feasibility of monitoring particle count from intubated subjects on mechanical ventilation after major surgery over a period of 72 h, investigating daily the impact of two different ventilation modes, VCV and PCV, as well as the particle pattern from the airways before, during and after a RM.

## Material and methods

### Animal preparation

The study was approved by the local Ethics Committee for Animal Research (Dnr 8401/2017). All animals received care according to the European Convention for the Protection of Vertebrate Animals used for Experimental and Other Scientific Purposes, as well as to the USA Principles of Laboratory Animal Care of the National Society for Medical Research, and the Guide for the Care and Use of Laboratory Animals, published by the National Academies Press (1996).

Six Swedish landrace pigs with a mean weight of 63 ± 1.4 kg were fasted overnight with free access to water. Premedication was performed with an intramuscular injection of Xylazine (Rompun® vet. 20 mg/ml; Bayer AG, Leverkusen, Germany; 2 mg/kg) mixed with ketamine (Ketaminol® vet. 100 mg/ml; Farmaceutici Gellini S.p.A., Aprilia, Italy; 20 mg/kg) in their stables, and a peripheral IV access was established in the earlobe. The pig was then transferred to the laboratory and placed in a supine position on the operating table. Oral intubation was performed using a 7.5-size endotracheal tube after anaesthesia induction with sodium thiopental (Pentothal; Abbott Laboratories, North Chicago, Illinois, USA) and pancuronium bromide (Pavulon; N.V. Organon, Oss, the Netherlands). Anaesthesia was maintained with a ketamine (Ketaminol® vet), midazolam (Midazolam Panpharma®, Oslo, Norway) and fentanyl (Leptanal®, Lilly, France) infusion. Fluid loss was compensated for by continuous infusion of Ringer’s acetate. Mechanical ventilation was established with a Siemens-Elema ventilator (Servo Ventilator 300, Siemens, Solna, Sweden).

The animals were carefully turned on the sides about 30° in each direction according to a schedule to reduce the risk of atelectasis.

### Abdominal surgery

A laparotomy was performed to mimic a clinical situation in an intensive care unit with mechanical ventilation on the subject with no previous lung injury, and all animals had a secondary temporary closure with negative pressure wound therapy (NPWT) in a standard way. A 30-cm-long midline incision was performed on each pig. The V.A.C.® Abdominal Dressing (KCI®, Inc., San Antonio, TX, USA) was used. The visceral protective layer was cut to an approximate size of 35 cm wide and 35 cm long, extending into the paracolic gutters on both sides. A layer of polyurethane Granu Foam was placed on top of the visceral protective layer between the edges of the wound. The wound was covered with a self-adhesive polyethylene drape, and a track pad was inserted through the drape (all from V.A.C., KCI, San Antonio, TX), and then connected to a continuous vacuum source with a negative pressure of − 120 mmHg. No manipulation of the abdominal organs was made.

### Mechanical ventilation and pulmonary recruitment manoeuvre

All animals had endotracheal tubes size 7.5, ventilator settings with tidal volume of 6 ml/kg, positive end-expiratory pressure of 5 cmH_2_O and end-inspiratory pressures < 25 cmH_2_O with an inspiratory to expiratory ratio (I:E) of 1:2. These settings remained unchanged during the study period. Particle outflow was measured using a modified PExA 2.0 instrument (PExA, Gothenburg, Sweden) each day during two different ventilation modes: VCV and PCV.

The animals were randomised into two different groups: one group received VCV before PCV (*n* = 3) and the other group received PCV before VCV (*n* = 3). Each animal was monitored during VCV and PCV for 1 h each per day. Before the collection period began for the second ventilation mode, there was an equilibration period of 30 min with the second ventilation mode. Twice daily, a RM was performed for 60 s at PEEP 10, 4 breath/min and I:E 2:1. Measurements were done 3 min before the RM, 1 min during the RM and 3 min after the RM.

### PExA measurements

The PExA 2.0 instrument (PExA, Gothenburg, Sweden) conducts measurements by optical particle counter and has been previously described in conjunction with mechanical ventilation. The instrument was connected to the outflow air of the mechanical respiratory circuit. The total accumulated number of particles from the airways was continuously measured by the PExA instrument during the two different ventilation modes each day. PExA measurements were made starting on day 1 until termination of mechanical ventilation on day 3. Measurements were made during each ventilation mode (VCV or PCV), each lasting 1 h in duration per day. Additionally, we monitored particle flow before, during and after RMs. The total particle count was measured for 3 min before the RM, for 1 min during the RM and for 3 min after the RM. Particles in the diameter range of 0.41–4.55 μm were measured by the PExA instrument. The particles are computationally divided into eight different size distributions according to their mean diameter. The PExA instrument does not take into count if the particles are proteins or lipids, endogenous or exogenous, the instrument measures only the particles diameter, no matter what the particles origin is. In this study, we only present the particles according to their mean diameter and not their physiological abilities. The mean diameter of the particles are as follows: particle 1, 0.48 μm; particle size 2, 0.59 μm; particle size 3, 0.75 μm; particle size 4, 0.98 μm; particle size 5, 1.22 μm; particle size 6, 1.67 μm; particle size 7, 2.52 μm; and particle size 8, 3.37 μm.

### Blood gases and haemodynamic parameters

All animals had a central venous catheter (CVC). The blood gases were drawn from the CVC and analysed in a standard way. Haemodynamic parameters were continuously recorded in a standard way.

### Experimental timeline

Mechanical ventilator settings were named as follows: VCV day 1 (VCV_1_), VCV day 2 (VCV_2_), VCV day 3 (VCV_3_), PCV day 1 (PCV_1_), PCV day 2 (PCV_2_) and PCV day 3 (PCV_3_).

### Calculations and statistics

Descriptive statistics, in the form of the number of patients, mean and the standard error of the mean (SEM) for the different parameters were analysed. The results are presented for the different parameters divided into the different groups. Statistically significant differences between the groups were tested using a paired Student *t* test. All statistical analyses were performed, using GraphPad Prism Software, CA, USA. Significance was defined as *p* < 0.001 (***), *p* < 0.01 (**), *p* < 0.05 (*), and *p* > 0.05 (not significant, n.s.).

## Results

### Animals

Pre-operative venous oxygen saturation (SvO_2_) at a FiO_2_ of 0.5 was 60.5 ± 6 kPa with a saturation of 98 ± 1%. Baseline mean blood pressure and pulse was 81 ±  3mmHg and 79 ± 12 beats per minute, respectively. No anatomical anomalies, signs of infection or malignancy were found in any of the animals at autopsy. One animal developed ARDS and was excluded and will be presented separately.

### Feasibility of the PExA method used in conjugation with mechanical ventilation

This study has been performed as a feasibility study. No adverse events (mild, moderate or severe) as airway leakage, signs of rebreathing, altered pressure levels and haemodynamic interferences were seen. Ventilator peak pressures and mean pressures along with FiO_2_ levels, venous blood gases, blood pressure, saturation and pulse are shown in Tables 1, 2 and 3. Interestingly, we have not detected any statistic significant or clinically significant changes during the 3 days.

**Table 1:** Hemodynamics parameters at the start and at the end of the daily measurements during volume-controlled ventilation (VCV)

	Start VCV	End VCV	Start VCV	End VCV	Start VCV	End VCV
	Day 1	Day 1	Day 2	Day 2	Day 3	Day 3
Blood gases						
ScvO2	61 ± 7	57 ± 6	77 ± 4	79 ± 3	82 ± 3	79 ± 3
pH	7.31 ± 0.01	7.32 ± 0.01	7.35 ± 0.02	7.33 ± 0.03	7.36 ± 0.04	7.37 ± 0.02
Bicarbonate	29 ± 1	30 ± 1	27 ± 1	27 ± 1	25 ± 2	27 ± 1
Base excess	6.3 ± 1.4	3.5 ± 0.6	3.9 ± 0.6	3.5 ± 0.6	1.1 ± 2.6	3.9 ± 0.9
Lactate	1.4 ± 0.3	1.2 ± 0.2	0.5 ± 0.1	0.5 ± 0.1	0.5 ± 0.1	0.6 ± 0.1
Mechanical ventilation						
Volume/minute	7840 ± 271	7840 ± 271	7840 ± 271	7840 ± 271	7840 ± 271	7840 ± 271
Breath/minute	20 ± 0	20 ± 0	20 ± 0	20 ± 0	20 ± 0	20 ± 0
PEEP	5 ± 0	5 ± 0	5 ± 0	5 ± 0	5 ± 0	5 ± 0
FiO2	0.5 ± 0	0.5 ± 0	0.5 ± 0	0.5 ± 0	0.5 ± 0	0.6 ± 0.2
Pressure (peak)	14 ± 1	15 ± 1	18 ± 2	17 ± 2	19 ± 3	20 ± 3
Pressure (mean)	7 ± 1	8 ± 1	8 ± 1	8 ± 1	8 ± 1	8 ± 1
Haemodynamics						
Pulse	77 ± 11	68 ± 8	72 ± 4	73 ± 6	86 ± 15	96 ± 20
Systolic BP	124 ± 7	111 ± 7	121 ± 6	119 ± 8	124 ± 10	127 ± 13
Diastolic BP	65 ± 7	70 ± 6	76 ± 5	73 ± 6	79 ± 9	85 ± 16
Mean BP	85 ± 5	83 ± 6	91 ± 5	88 ± 6	94 ± 9	99 ± 15
Saturation	97 ± 1	96 ± 1	95 ± 1	95 ± 1	95 ± 1	94 ± 2

**Table 2:** Hemodynamics parameters at the start and at the end of the daily measurements during pressure-controlled ventilation (PCV)

	Start PCV	End PCV	Start PCV	End PCV	Start PCV	End PCV
	Day 1	Day 1	Day 2	Day 2	Day 3	Day 3
Blood gases						
ScvO2	58 ± 7	56 ± 7	78 ± 2	76 ± 5	87 ± 4	80 ± 4
pH	7.34 ± 0.02	7.35 ± 0.01	7.32 ± 0.02	7.33 ± 0.02	7.36 ± 0.01	7.37 ± 0.03
Bicarbonate	30 ± 1	29 ± 1	27 ± 1	27 ± 1	27 ± 1	27 ± 1
Base excess	6.7 ± 0.5	7 ± 1.2	3.3 ± 0.6	4 ± 0.7	3.2 ± 0.9	4 ± 0.9
Lactate	1.3 ± 0.1	1.4 ± 0.2	0.5 ± 0.1	0.5 ± 0.1	0.5 ± 0.1	0.5 ± 0.1
Mechanical ventilation						
Volume/minute	8000 ± 294	7840 ± 271	7840 ± 271	7840 ± 271	7840 ± 271	7840 ± 271
Breath/minute	20 ± 0	20 ± 0	20 ± 0	20 ± 0	20 ± 0	20 ± 0
PEEP	5 ± 0	5 ± 0	5 ± 0	5 ± 0	5 ± 0	5 ± 0
FiO2	0.5 ± 0	0.5 ± 0	0.5 ± 0	0.5 ± 0	0.6 ± 0.1	0.6 ± 0.1
Pressure (peak)	15 ± 1	15 ± 1	16 ± 1	16 ± 1	19 ± 2	18 ± 2
Pressure (mean)	8 ± 1	8 ± 1	8 ± 1	8 ± 1	8 ± 1	8 ± 1
Haemodynamics						
Pulse	81 ± 15	72 ± 9	74 ± 7	71 ± 3	101 ± 20	93 ± 16
Systolic BP	108 ± 8	111 ± 11	121 ± 9	116 ± 6	132 ± 13	115 ± 11
Diastolic BP	62 ± 8	61 ± 7	75 ± 7	68 ± 7	79 ± 17	63 ± 9
Mean BP	77 ± 7	77 ± 7	90 ± 7	84 ± 6	96 ± 15	80 ± 9
Saturation	96 ± 1	96 ± 1	95 ± 1	96 ± 1	92 ± 3	93 ± 2

**Table 3:** Hemodynamics parameters before and after recruitement manoeuvres (RM) during the daily measurements

	Before	After	Before	After	Before	After
	Day 1	Day 1	Day 2	Day 2	Day 3	Day 3
Blood gases						
ScvO2	58 ± 4	48 ± 5	82 ± 2	79 ± 1	82 ± 2	78 ± 3
pH	7.34 ± 0.01	7.33 ± 0.01	7.33 ± 0.01	7.32 ± 0.01	7.37 ± 0.01	7.35 ± 0.02
Bicarbonate	29 ± 1	30 ± 1	27 ± 1	27 ± 1	27 ± 1	26 ± 1
Base excess	7.0 ± 0.5	7.3 ± 0.5	3.4 ± 0.3	3.2 ± 0.4	3.5 ± 0.5	2.5 ± 1.3
Lactate	7.1 ± 1.0	1.2 ± 0.2	0.4 ± 0.1	0.4 ± 0.1	0.5 ± 0.1	0.5 ± 0.1
Mechanical ventilation						
Volume/minute	7840 ± 171	7840 ± 171	7840 ± 171	7840 ± 171	8000 ± 173	8000 ± 173
Breath/minute	20 ± 0	20 ± 0	20 ± 0	20 ± 0	20 ± 0	20 ± 0
PEEP	5 ± 0	5 ± 0	5 ± 0	5 ± 0	5 ± 0	5 ± 0
FiO2	0.5 ± 0	0.5 ± 0	0.5 ± 0	0.5 ± 0	0.5 ± 0	0.5 ± 0
Pressure (peak)	15 ± 1	14 ± 1	17 ± 1	16 ± 1	16 ± 1	16 ± 1
Pressure (mean)	8 ± 1	8 ± 1	8 ± 1	8 ± 1	8 ± 1	8 ± 1
Haemodynamics						
Pulse	71 ± 6	67 ± 5	79 ± 5	78 ± 2	73 ± 5	70 ± 4
Systolic BP	112 ± 5	112 ± 7	123 ± 4	121 ± 4	118 ± 8	114 ± 7
Diastolic BP	64 ± 5	68 ± 5	76 ± 4	74 ± 4	71 ± 6	67 ± 7
Mean BP	80 ± 4	83 ± 5	92 ± 4	90 ± 5	87 ± 6	83 ± 7
Saturation	96 ± 1	96 ± 1	95 ± 1	95 ± 1	96 ± 1	96 ± 1

### Effects of mechanical ventilation on total particle count from the airways

**Fig. 1:**
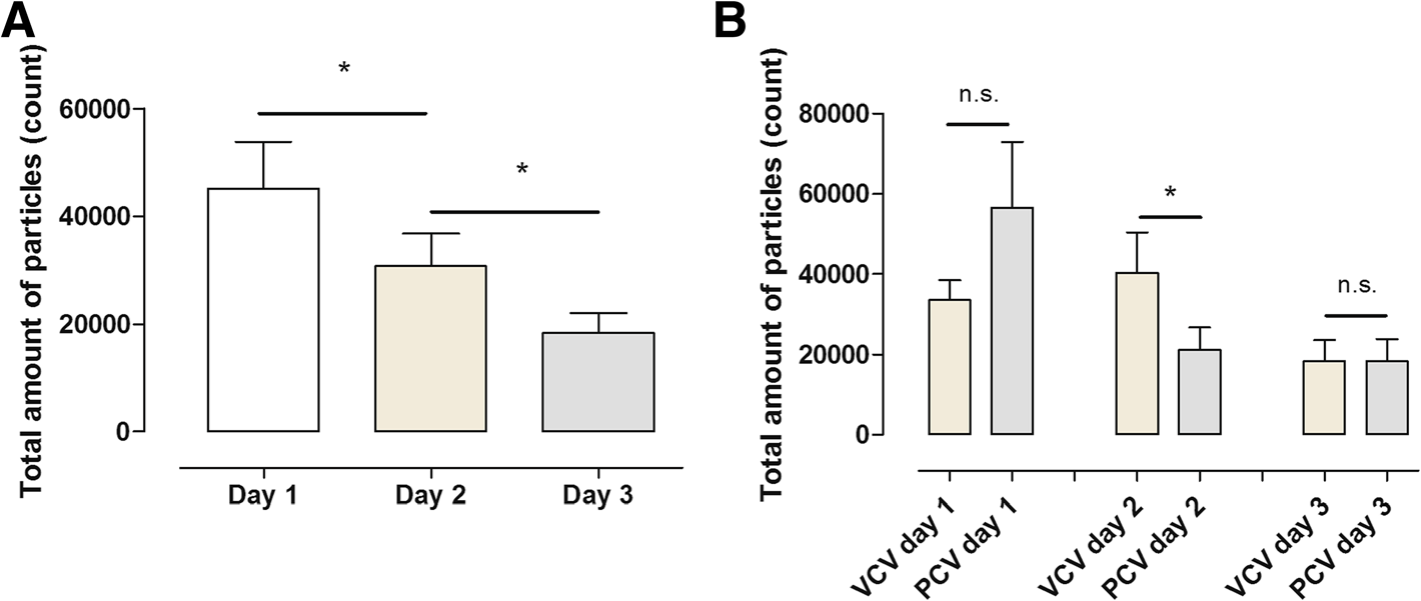
The figure shows the total accumulated particle count measured by a customised PExA instrument during volume-controlled ventilation (VCV) and pressure-controlled ventilation (PCV) during three consecutive days (*n* = 5) (**a**). In **b**, VCV and PCV are divided into separate groups. Note the significant increase in total particle count on day 2

VCV and PCV were measured on a daily basis from day 1 until day 3 in all animals. On day 1, the total particle count was 45,063 ± 8775; on day 2, the total particle count was 30,749 ± 6033; and on day 3, the total particle count was 18,409 ± 3693. Comparing day 1 and day 2, a significant difference was found (*p* = 0. 0274). A significant decrease in particle flow was seen on day 3 compared to day 2 (*p* = 0.0246) (Fig. 1). One animal was excluded due to the development of clinical signs of ARDS, and the particle count from the airways in this animal was much higher than in the other animals.

### Effects of volume-controlled ventilation (VCV) and pressure-controlled ventilation (PCV)

VCV was compared to PCV from day 1 until day 3. On day 1, the total particle count was 33,562 ± 4951 during VCV_1_ and 56,564 ± 16,468 during PCV_1_ (*p* = 0.1779); on day 2, the total particle count was 40,260 ± 10,097 during VCV_2_ and 21,238 ± 5625 during PCV_2_ (*p* = 0.0184); and on day 3, the total particle count was 18,343 ± 5347 during VCV_3_ and 18,497 ± 5418 during PCV_3_ (*p* = 0.5977) (Fig. 1).

### Particle distribution during VCV and PCV

**Fig. 2:**
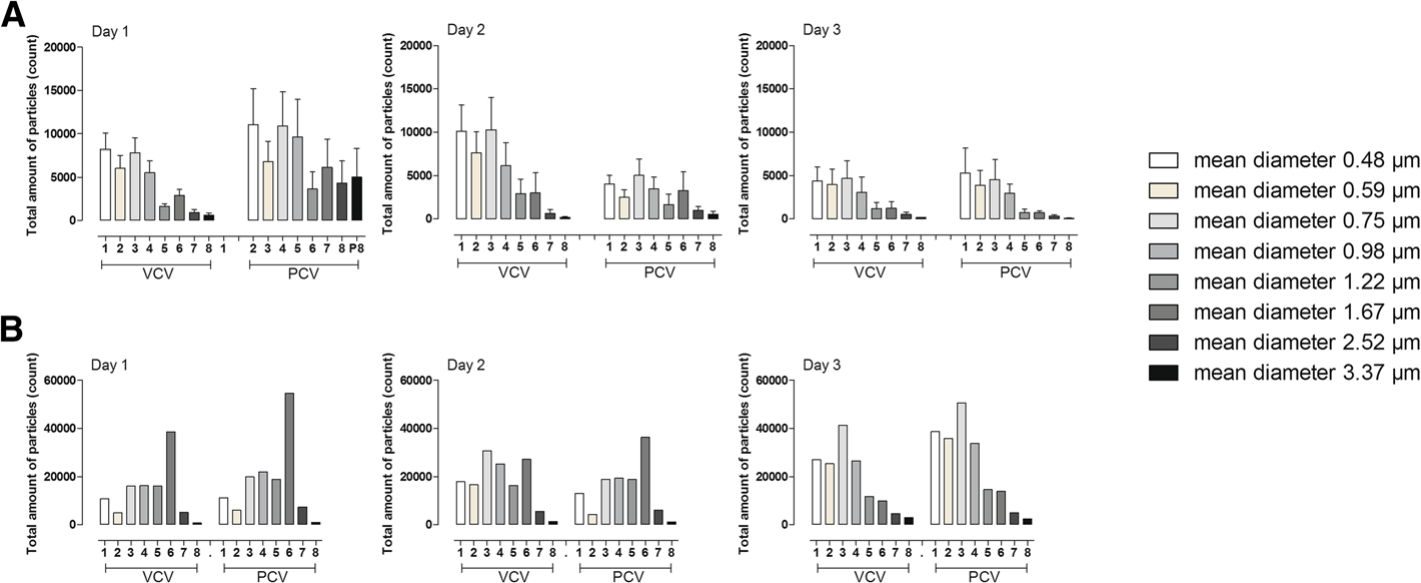
Particles in the diameter range of 0.41–4.55 μm were measured by the customised PExA instrument. The particles are computationally divided by the optical particle counter in the PExA device into eight different size distributions with a mean diameter of particle size 1, 0.48 μm; particle size 2, 0.59 μm; particle size 3, 0.75 μm; particle size 4, 0.98 μm; particle size 5, 1.22 μm; particle size 6, 1.67 μm; particle size 7, 2.52 μm; and particle size 8, 3.37 μm. The figure displays different particle size distributions during volume-controlled ventilation (VCV) and pressure-controlled ventilation (PCV) in five animals during three consecutive days (**a**). One animal developed ARDS on day 3. Note how particles within size 6 were increased during day 1–2. On day 3, when the animal developed clinical signs of ARDS, a similar particle size pattern with the other five animals was seen (**b**)

The total particle count from the airways is divided by the optical particle counter into eight different size distributions according to particle size where particle size 1 is the smallest and particle size 8 is the largest. The results of particle distribution from VCV and PCV modes during the three subsequent days are shown in Fig. 2. One animal developed severe ARDS on day 3 and had a different particle pattern from the airways from day 1 with significantly increased particle size 6. Interestingly, on day 3, when the animal developed ARDS, the particle size 6 decreased. The animal that developed clinical signs of ARDS was excluded and is presented separately (Fig. 2).

### Recruitment manoeuvres (RM)

**Fig. 3:**
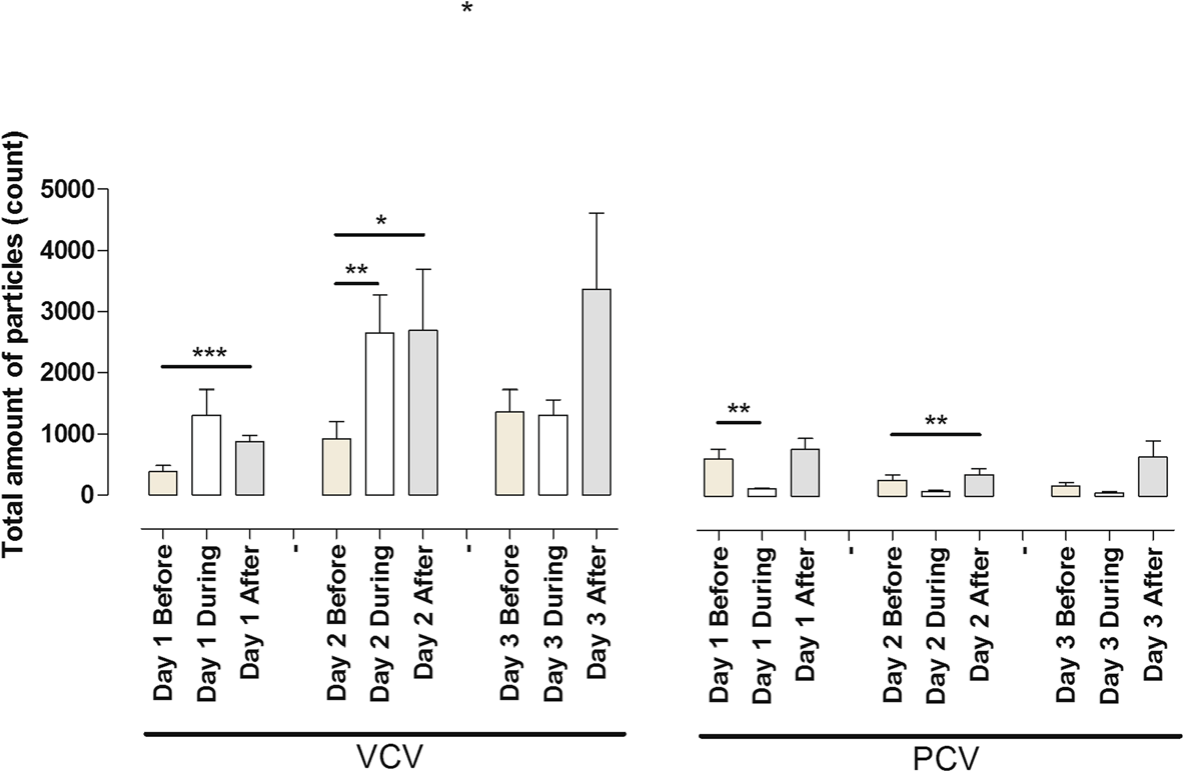
All animals underwent recruitment manoeuvre (RM) twice daily starting on day 1 until day 3. The total accumulated particle count was measured by PExA 3 min before RM, 1 min during the RM and 3 min after RM. The figure shows the total particle count before, during and after RM for three consecutive days using volume-controlled ventilation (VCV) and pressure-controlled ventilation (PCV)

The total particle count was measured 3 min before the RM, 1 min during the RM and 3 min after the RM. A RM was performed daily using VCV mode resulting in a total particle count of 379 ± 106 before, 1303 ± 427 during and 873 ± 103 after recruitment day 1. Comparing the particle flow before and after RM, a significant difference was observed (*p* = 0.0001). Comparing the particle flow before and during RM (*p* = 0.0746), no significant difference was found. The total particle count was 918 ± 281 before, 2652 ± 6 during and 2692 ± 995 after recruitment day 2. Comparing the particle flow before and after RM, a significant difference was observed (*p* = 0.0476). Comparing the particle flow before and during RM, a significant increase could also be observed (*p* = 0.0161). On day 3, the total particle count was 1357 ± 366 before, 1309 ± 251 during and 3359 ± 1252 after recruitment day 3. Comparing the particle flow before and after RM, no significant difference was observed (*p* = 0.0920). Comparing the particle flow before and during RM, no significant difference was observed (*p* = 0.8577) (Fig. 3).

A RM was performed daily using PCV mode, and the total particle count was 737 ± 186 before, 146 ± 22 during and 923 ± 212 after recruitment day 1. Comparing the particle flow before and after RM, no significant difference was observed (*p* = 0.0563). Comparing the particle flow before and during RM, a significant decrease was observed (*p* = 0.0083). The total particle count was 321 ± 105 before, 104 ± 22 during and 433 ± 117 after recruitment day 2. Comparing the particle flow before and after RM, a significant increase was observed (*p* = 0.0157). Comparing the particle flow before and during RM, no significant difference could be observed (*p* = 0.0520). On day 3, the total particle count was 208 ± 59 before, 73 ± 21 during and 771 ± 312 after recruitment. Comparing the particle flow before and after RM, no significant difference was observed (*p* = 0.0565). Comparing the particle flow before and during RM (*p* = 0.0824), no significant difference was observed (Fig. 3).

**Fig. 4:**
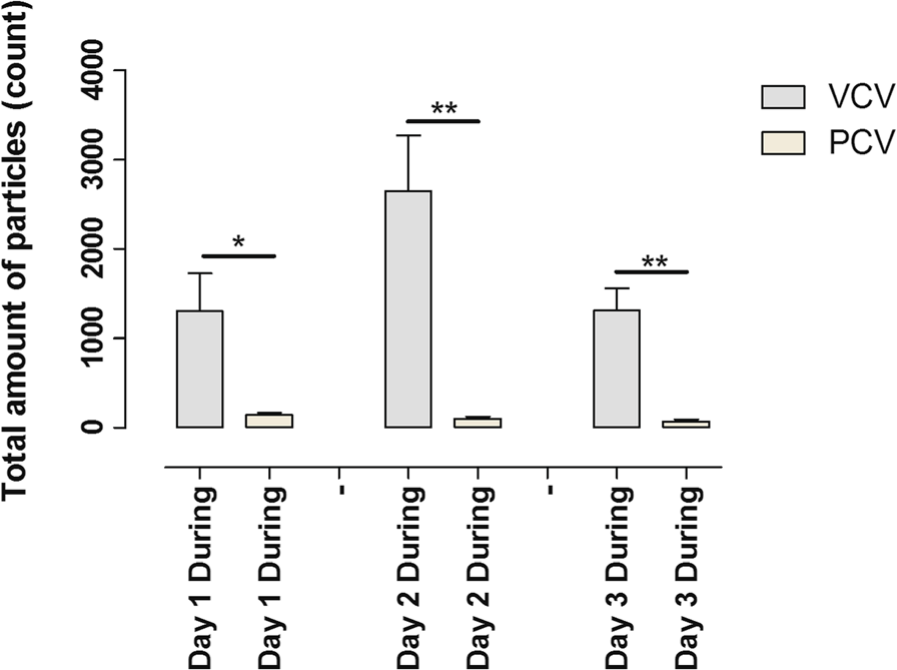
A recruitment manoeuvre (RM) was performed on all animals twice daily starting on day 1 until day 3. The total accumulated particle count was measured by PExA during the RM for 1 min. The figure shows the total particle count during RM during three consecutive days comparing volume-controlled ventilation (VCV) and pressure-controlled ventilation (PCV). Note the significant differences in total particle count during VCV and PCV before the actual RM

Comparing particle flow for VCV with PCV before and after RM, a significant difference was found on day 1 (*p* = 0.0185) but not on day 2 (*p* = 0.0584) or day 3 (*p* = 0.3013). Particle flow during RM found significant differences on all three days: day 1 VCV vs day 1 PCV (*p* = 0.0232), day 2 VCV vs day 2 PCV (*p* = 0.0016) and day 3 VCV vs day 3 PCV (*p* = 0.0112) (Fig. 4).

### Fluid balance

**Fig. 5:**
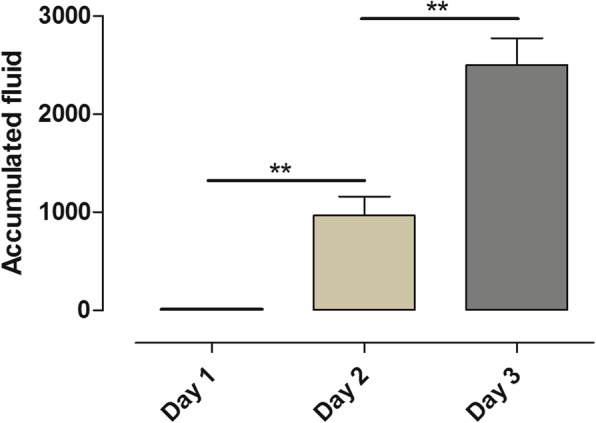
The animals’ total accumulated fluid balance during three consecutive days. Note how the total accumulated fluid balance significantly increased over the 3 days

The fluid balance was measured daily. On day 1, the fluid balance was 0 ± 0 ml; on day 2, the fluid balance was 968 ± 193 ml; and on day 3, the fluid balance was 2498 ± 275 ml. Statistical significance was found comparing day 1 and day 2 (*p* = 0.0014) and day 2 and day 3 (*p* = 0.0042) (Fig. 5).

### Blood gases, haemodynamics and mechanical ventilation settings

The blood gases, haemodynamics and mechanical ventilation settings during the different ventilation settings and during the different days are shown in Tables 1 and 2. Measurements were taken at the start and at the end of each ventilation mode. In Table 3, the same parameters are shown and were taken 3 min before pulmonary recruitment and 3 min after RM. Fluid was given to maintain vital signs and an adequate urine output. All animals were stable during all measurements, and no significant changes in blood gases, haemodynamics or in mechanical ventilation settings could be found.

## Discussion

The main techniques to monitor patients’ airways during mechanical ventilation are by pressure, volume and airflow. Noninvasive online analysis of different particle flow from the airways might provide an additional tool for monitoring the patient during mechanical ventilation. We have recently shown in a lung transplantation animal model that different ventilation modes resulted in different particle flow from the airways in vivo, post mortem and during ex vivo lung perfusion [8]. Exhaled breath particles (EBPs) are believed to originate from the respiratory tract lining fluid (RTLF). The RTLF consists of heterogenous group of substances covering the epithelial wall of the airways and has a different composition in different parts of the airways. The exact origin and formation of all particles in the airway are not fully clear, but what is known is that surfactant A is a large component produced in the alveoli by type II alveolar cells. Surfactant A’s composition is of phospholipids, proteins, peptides and nucleic acids. The RTLF also contains glycoproteins and mucins predominantly from the conducting airways. Studies have shown that an altered composition of the RTLF might reflect different airway diseases [11, 16–19]. Since the animals are intubated and on mechanical ventilation, the PExA instrument is not in contact with particles originating from the mouth and nasopharynx, but still, some of the conducting airways are below the endotracheal tube.

Mechanical ventilation is used in several clinical conditions in an intensive care unit. Lung-protective ventilation strategies with the use of low tidal volume and low to moderate PEEP have been shown to improve outcomes in patients with or at risk for ARDS [20], but also in patients without pre-existing lung injury, using low tidal volume and moderate PEEP has also proven beneficial [21]. Mechanical ventilation, particularly high tidal volume and the use of very low PEEP or no PEEP at all, increases the collapse of the distal airways and the alveoli, and this is known to have a negative impact on lung function not only in ARDS patients but in patients with healthy lungs. In order to prevent collapse of the airways, ARDSnet suggests different levels of PEEP, according to the open lung concept, that have shown favourable outcome with improved pulmonary function without causing further harm [22]. In the present study, we follow the recommendations from ARDSnet by using low tidal volumes and moderate PEEP and studying if there is any difference in particle flow when comparing two commonly used ventilation modes, VCV and PCV. All animals underwent a laparotomy on day 1 before any PExA measurements were made to mimic a clinical situation in an intensive care unit in subjects with no previous lung injury, and all animals thereafter were studied for three consecutive days. All animals were fully anaesthetised, and no animal drew their own breath.

### The PExA instrument is safe to use conjugation with mechanical ventilation

This study has been performed as a feasibility study. No adverse events (mild, moderate or severe) as airway leakage, signs of rebreathing, altered pressure levels and haemodynamic interferences were seen. As shown in Tables 1, 2 and 3, we have not detected any significant changes during the 3 days. Furthermore, this study has not shown any harmful effects on the subjects either in ventilator measurements or haemodynamic measurements; thereby, we believe this technique can safely be used in conjugation with mechanical ventilation. The method has the potential to shed new light on the physiological changes within the lung during mechanical ventilation.

### Decreased particle flow in relation to increased fluid levels

In all the animals, we noticed a stepwise significant decrease in total particle count during the 3 days (Fig. 1a). In studies with ARDS patients, it has been proven that excessive fluid overload leads to poorer outcome [23, 24]. Over the course of the 3 days, the animals in this study showed an increase in fluid levels as well as a decrease in total particle count (Figs. 1a and 5). We speculate that the decrease in total particle count may be due to the fluid overload in the alveoli.

### Lung response to different ventilation modes depending on time on mechanical ventilation

Comparing the total particle count for VCV and PCV day by day during the 3 days, counting the particle flow from day 1 PCV had a higher particle count than VCV, but not significant. Interestingly, a similar reproducible pattern with higher particle flow for PCV compared to VCV has been described in a previous study [8]. On day 2, a significantly higher total particle count could be observed for VCV compared to PCV. Interestingly, on day 3, there was no difference between the two modes (Fig. 1b). During all the days, there were no changes in the commonly used indicators of lung function such as pressure levels, FiO_2_ levels or the animals’ vital parameters. These findings may show that the different ventilation modes may have a greater impact and generate a different biochemical environment in the RTLF and EBP than currently understood.

### Altered composition in RTLF may correlate to airway disease

The PExA instrument has the possibility to both measure particle count from the airways and divide the different particles according to eight different sizes, from 0.48 to 3.37 μm in mean diameter. By studying the particle patterns according to size, it might give us additional information on the physiology of the small airways and the impact of mechanical ventilation. The composition between the eight different particle sizes had the same pattern when comparing the two different modes (Fig. 2a). In the animal that was excluded due to developing severe acute clinical signs of ARDS on day 3, but not on day 1 or day 2, a very different particle pattern was observed compared to the other five animals, when studying the different size distributions divided by the PExA instrument. We could observe an increase of total particle count in both VCV and PCV mode for this animal compared to the others during all measurements on all the days. Interestingly, the pattern of particles size 6 with a mean diameter of 1.67 μm stood out in particular, both in VCV and PCV compared to the other animals (Fig. 2b). To focus on particle size 6, it had a severely increased particle count on day 1 and 2 when there were no clinical signs of ARDS, but on day 3, when the animal developed acute clinical signs of ARDS, particle size 6 was severely reduced compared to previous days, and the particle pattern for size 1–8 was similar to the other animals. We find this change in the composition of the EBP very interesting as it may be an early sign of developing lung injury.

### Recruitment manoeuvre (RM) opens up the alveoli, but the effect is short lived

We performed a RM in all animals, every day in both ventilation modes. Measurement was done for 3 min before the RM, during 1 min of the RM and for 3 min after the RM. We could see an increase in the total particle count after the RM compared to before the RM in both VCV and PCV. We believe that the reason for these results is that the actual RM opens up the alveoli’s and thereby a higher particle count (Fig. 3). We could also see that this effect of higher particle count was fairly short lived, and the effect was predominantly over within 2–3 min.

### The lung physiology during RM is different depending on ventilation mode

During the RM, a difference was observed in total particle count depending on what ventilation mode was used. In VCV, there was an increased total particle count during the RM compared to before the RM. In PCV, on the other hand, there was instead a decreased total particle count during the RM compared to before the RM. The total particle count during RM was higher in VCV compared to PCV during all the days (Fig. 4). We interpret these findings that increasing the PEEP to 10 does not have the same impact on the small airways for VCV and PCV, and we speculate that a RM during VCV leads to increased opening and closing of the small airways compared to PCV.

## Limitations

This study is an experimental study and has been performed under controlled conditions in a laboratory setting in a small healthy cohort without underlying lung injury. The study has been performed as a feasibility study, and therefore, we have chosen to study a limited amount of ventilatory settings, and we have focused on two different modes and a gentle RM.

Changing tidal volumes, respiratory rate or I:E ratios might have given more evolving data, but as this is the first study in its kind, we wanted to focus on feasibility and safety. All pigs have been in a supine position throughout the study period, and that might have affected the result since this position for this long period of time may promote atelectasis. The pigs were turned on their side about 30° according to a schedule to reduce the risk of atelectasis, and due to abdominal surgery and vacuum closure, another position would be very difficult to achieve.

The PExA instrument only detects particle in the diameter range of 0.41–4.55 μm and there can be particles outside this range. The majority of particles originating from RTLF are presumably within this range. We have not studied if any particles have been deposited on the inside lining of the endotracheal tube or other parts of the respiratory circuit. A proportionate deposition of particles on the entire respiratory circuit between the different subjects could be assumed, since all the subjects were exposed to the same length of the respiratory circuit.

The particles from the airways were collected onto a membrane for subsequent biochemical analyses. On day 1, we were able to collect significantly more mass of particles (mean size 24.8 ng) than on days 2 and 3; however, unfortunately, it was not sufficient for analyses with our current methods. We are currently trying to improve the analysing techniques. In spite of this, these findings may provide further understanding on the physiology of the small airways and the impact of mechanical ventilation.

## Conclusions

This study has primarily been done as a feasibility study to assess the safety and useability of the PExA technique. We believe this study has been rudimental in proving that the PExA technique is safe to use in conjunction with mechanical ventilation both as single use but also repeatedly over days. The results indicate that different ventilation modes, such as VCV and PCV, may have a greater impact and generate a different biochemical environment in the small airways than currently understood. The findings during recruitment manoeuvres indicate an opening of the small airways, but the effect is short lived. We have also showed that VCV and PCV may affect the lung physiology differently during recruitment manoeuvres. During the 3 days, all animals showed a stepwise decrease in particle count and at the same time increased fluid levels, which might indicate a relationship between the particle count and the fluid levels, i.e., the more fluid overload, the less particle count. The animal that developed ARDS showed a different particle composition compared to the other animals, which might correlate to an alteration of the RTLF. Interestingly, we could detect differences in particle flow without any significant changes in ventilator pressure levels, FiO2 levels or the animals’ vital parameters, and our interpretation is that this technique might very well give more detailed information about the impact of mechanical ventilation.

## Abbreviations

ALI: Acute lung injury
ARDS: Acute respiratory distress syndrome
CVC: Central venous catheter
EBP: Exhaled breath particles
I:E: Inspiratory to expiratory ratio
PCV: Pressure-controlled ventilation
PEEP: Positive end-expiratory pressure
PExA: Particles in exhaled air
RM: Recruitment manoeuvre
RTLF: Respiratory tract lining fluid
SEM: Standard error of the mean
SvO_2_: Venous oxygen saturation
VCV: Volume-controlled ventilation
VILI: Ventilator-induced lung injury
